# Historical Biobanks in Breast Cancer Metabolomics— Challenges and Opportunities

**DOI:** 10.3390/metabo9110278

**Published:** 2019-11-13

**Authors:** Torfinn S. Madssen, Maria D. Cao, Arne V. Pladsen, Lars Ottestad, Kristine K. Sahlberg, Tone F. Bathen, Guro F. Giskeødegård

**Affiliations:** 1 Department of Circulation and Medical Imaging, Norwegian University of Science and Technology, 7491 Trondheim, Norway; maria.d.cao@hiof.no (M.D.C.); tone.f.bathen@ntnu.no (T.F.B.); guro.giskeodegard@ntnu.no (G.F.G.); 2 Department of Cancer Genetics, Institute for Cancer Research, Oslo University Hospital, 0310 Oslo, Norway; Arne.Pladsen@rr-research.no (A.V.P.); larsottestad@gmail.com (L.O.); kristine.sahlberg@vestreviken.no (K.K.S.); 3 Department of Oncology, Østfold Hospital Trust, 1714 Kalnes, Norway; 4 Department of Research and Innovation, Vestre Viken Hospital Trust, 3004 Drammen, Norway

**Keywords:** metabolomics, breast cancer, tissue, biobank, NMR

## Abstract

*Background:* Metabolomic characterization of tumours can potentially improve prediction of cancer prognosis and treatment response. Here, we describe efforts to validate previous metabolomic findings using a historical cohort of breast cancer patients and discuss challenges with using older biobanks collected with non-standardized sampling procedures. *Methods:* In total, 100 primary breast cancer samples were analysed by high-resolution magic angle spinning magnetic resonance spectroscopy (HR MAS MRS) and subsequently examined by histology. Metabolomic profiles were related to the presence of cancer tissue, hormone receptor status, T-stage, N-stage, and survival. RNA integrity number (RIN) and metabolomic profiles were compared with an ongoing breast cancer biobank. *Results:* The 100 samples had a median RIN of 4.3, while the ongoing biobank had a significantly higher median RIN of 6.3 (*p* = 5.86 × 10^−7^). A low RIN was associated with changes in choline-containing metabolites and creatine, and the samples in the older biobank showed metabolic differences previously associated with tissue degradation. The association between metabolomic profile and oestrogen receptor status was in accordance with previous findings, however, with a lower classification accuracy. *Conclusions:* Our findings highlight the importance of standardized biobanking procedures in breast cancer metabolomics studies.

## 1. Introduction

Breast cancer is a highly heterogenous disease ranging from localized curable disease with minor impact on life expectancy to incurable metastatic disease with a poor prognosis. Treatment and prediction of prognosis are guided by anatomical and molecular characteristics of the tumour. However, current methods are not sufficiently accurate for predicting treatment benefit. Treatment is currently guided by clinical stage, expression of hormone receptors and human epidermal growth factor receptor 2 (HER2), and the proliferation marker Ki-67. Gene expression analyses have shown that breast cancer can be divided into intrinsic subtypes of prognostic value, with further stratification based on protein expression and metabolism being studied [1,2,3,4]. 

Tissue metabolism has also been associated with several prognostic and predictive factors in breast cancer, such as hormone receptor status [5] and treatment response [6,7]. We have previously suggested lactate and glycine as prognostic biomarkers in oestrogen receptor positive (ER+) breast cancer [7,8], and have further shown that breast cancer may be divided into metabolic subtypes based on the metabolomic profile of the tumour [3].

Analysis of samples from historical biobanks provides several research opportunities, including increased availability of tissue samples, and assessment of long-term survival. Further, the validation of previous findings in an independent cohort is a necessary step if they are to be used clinically. In this study, we used a historical biobank, OsloVal, consisting of surgical breast cancer biopsies gathered at Oslo University Hospital, Norway, between 1983 and 1997, and compared it with biopsies from Oslo2, an ongoing biobank. Because of the age of this biobank and the fact that a subset of it was not sampled or stored according to modern protocols, its suitability for metabolomic analyses was uncertain. This short communication describes our efforts to reproduce previous metabolomic findings in a historical biobank and highlights several issues with working with such samples. 

## 2. Results

### 2.1. Tissue Integrity

**Figure 1:**
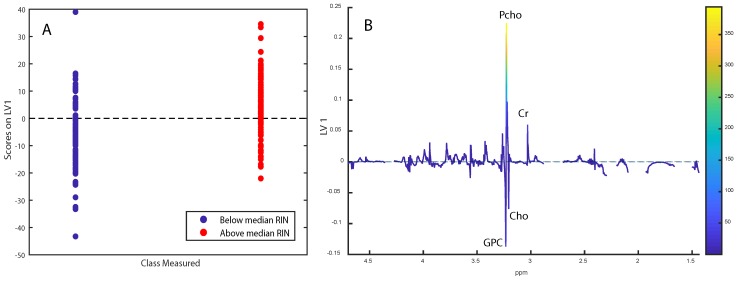
(**A**,**B**) OPLS-DA score- and loading plots for separating samples with RIN above the median from below the median. Labeled metabolites:glycerophosphocholine (GPC), phosphocholine (Pcho), choline (Cho), creatine (Cr). The loadings are colored according to VIP-scores.

The 100 samples in the OsloVal cohort were collected from two different time periods, 1983–1989 (*n* = 83) and 1994–1997 (*n* = 17). The median RNA integrity number (RIN) for samples collected after 1994 was 6.4, while samples collected from 1983–1989 had a median RIN of 4.0 (*p* = 0.077). The median RIN of the ongoing biobank, Oslo2 (*n* = 395, collected between 2006–2009), was 6.3. This was significantly higher than the RIN of the older samples of OsloVal (*p* < 0.001), but not significantly different from samples collected after 1994 (*p* = 0.78). When combining the OsloVal and Oslo2 data, OPLS-DA correctly classified samples as above or below median RIN with an accuracy of 64.3% (*p* < 0.001). The score and loading plots showed that the main discriminating metabolites were choline-containing compounds and creatine, with low-RIN-samples having higher levels of glycerophosphocholine (GPC) and choline and lower levels of creatine and phosphocholine (Pcho) (Figure 1A,B).

### 2.2. Histopathology and Immunohistochemistry

To confirm the presence of tumour tissue and the ER-status of the analysed samples, histopathology was performed on the OsloVal samples after HR MAS MRS. Out of the 100 samples, two were excluded because of indeterminate oestrogen receptor status due to cytoplasmic staining, and two samples were lost during preparation. In total, 22 samples did not contain tumour tissue, 21 of which were from 1983–1989. The clinical characteristics of the remaining 74 patients are given in Table 1. After reassessing ER status, 61 out of 74 samples retained their original classification collected from patient journals. In the 58 samples from the older subset of OsloVal in which receptor status was determined using dextran-coated charcoal assay, three changed status from positive to negative and three changed from negative to positive. In the 16 most recent OsloVal samples, four changed status from positive to negative and two changed from negative to positive.

### 2.3. Metabolic Comparison of OsloVal and Oslo2

**Figure 2:**
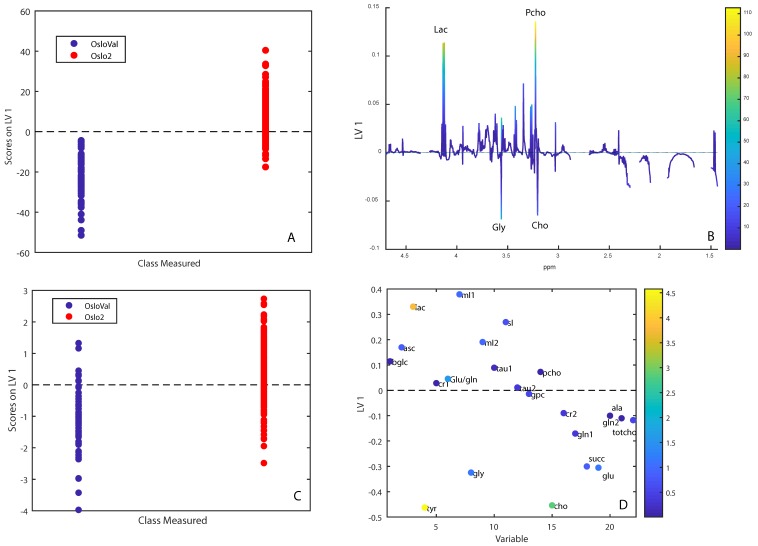
OPLS-DA score- and loading plots for separating samples from OsloVal and Oslo2. (**A**,**B**) show results from using spectral data. (**C**,**D**) show results using metabolite integration and after removing high-lipid samples. Variables in (**D**) are colored by VIP-score. Labeled metabolites: beta-glucose (bglc), ascorbate (asc), lactate (lac), tyrosine (tyr), creatine (cr1), glutamate/glutamine (glu/gln), myo-inositol (mI1), glycine (gly), myo-inositol (mI2), taurine (tau1), glycerophosphocholine (gpc), phosphocholine (pcho), choline (cho), creatine (cr2), glutamine (gln1), succinate (succ), glutamate (glu), glutamine (gln2), alanine (ala), total choline (totcho).

The metabolomic profiles of the two cohorts could be significantly discriminated using OPLS-DA, achieving a classification accuracy of 93.9%. The score and loading plots (Figure 2A,B) indicated that higher full width half maximums and peak shift for some peaks in OsloVal influenced the comparison. The FWHM of NMR peaks reflects homogeneity of the magnetic field, where high FWHM values result from inhomogeneities in the field.

In order to minimize differences due to shimming, shift correction, and lipid content, all samples in OsloVal in which lipids comprised more than 95% of the spectral area were removed (11 samples collected from 1983–1989, and two samples collected from 1994–1997) and normalized integrals from the processed spectra were used as model input instead of spectral data. The two cohorts could then be discriminated with an accuracy of 77.1% (*p* < 0.001). The score and loading plots from integrals show that samples from Oslo2 had higher levels of lactate, ascorbate, myo-inositol, and scyllo-inositol, while the OsloVal samples had higher levels of choline, glycine, tyrosine, glutamate, and succinate (Figure 2C,D).

### 2.4. Metabolomic Analyses in OsloVal

**Figure 3:**
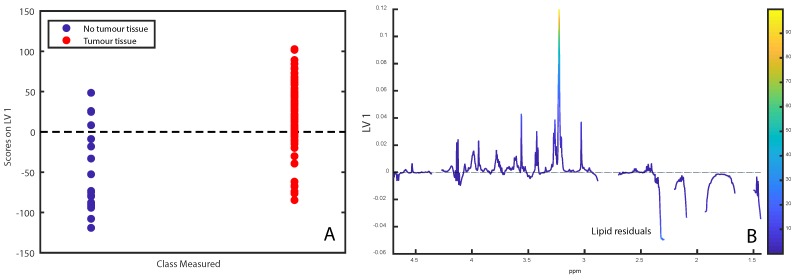
(**A**,**B**) OPLS-DA score- and loading plots for separating tumour tissue from non-involved tissue. Loadings are colored according to VIP-score.

OPLS-DA correctly classified samples according to tumour cell content with a classification accuracy of 74.6% (*p* < 0.001, Figure 3A,B). The score- and loading plots showed that samples with tumour cells had lower levels of lipid residuals compared with samples without tumour cells.

**Figure 4:**
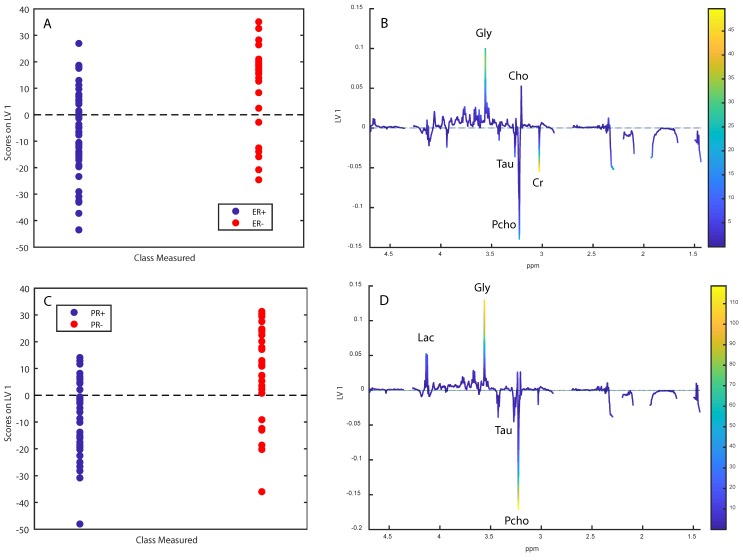
Score- and loading plots for prediction of oestrogen receptor status (**A**,**B**) and progesterone receptor status (**C**,**D**) respectively. Labeled metabolites are: Glycine (Gly), lactate (Lac), taurine (Tau), phosphocholine (Pcho), glycerophosphocholine (GPC), choline (Cho), and creatine (Cr). The loadings are colored according to Variable Importance in Projection (VIP) scores.

OPLS-DA classified OsloVal-samples according to ER-status with a sensitivity and specificity of 57.6% and 68.6%, respectively, giving a classification accuracy of 63.1% (Figure 4A,B, *p* = 0.008). The score and loading plots show that ER+ samples had higher levels of creatine, taurine and phosphocholine, while ER samples had higher levels of glycine and choline. Progesterone receptor status could be predicted from the metabolomic profiles with a sensitivity and specificity of 63.0% and 60.2%, respectively, producing a classification accuracy of 61.9% (Figure 4C,D, *p* = 0.011). PR+ samples had higher levels of taurine and Pcho, while PR- tumours had higher levels of lactate, glycine, GPC and choline.

OPLS-DA correctly classified samples according to tumour size (T1 or higher than T1) with a low accuracy of 59.0%. Furthermore, samples could not be classified according to lymph node spread (accuracy = 55.5%) or between histopathological grades (grade 1 or 2 vs. grade 3, accuracy = 52.7%).

**Figure 5:**
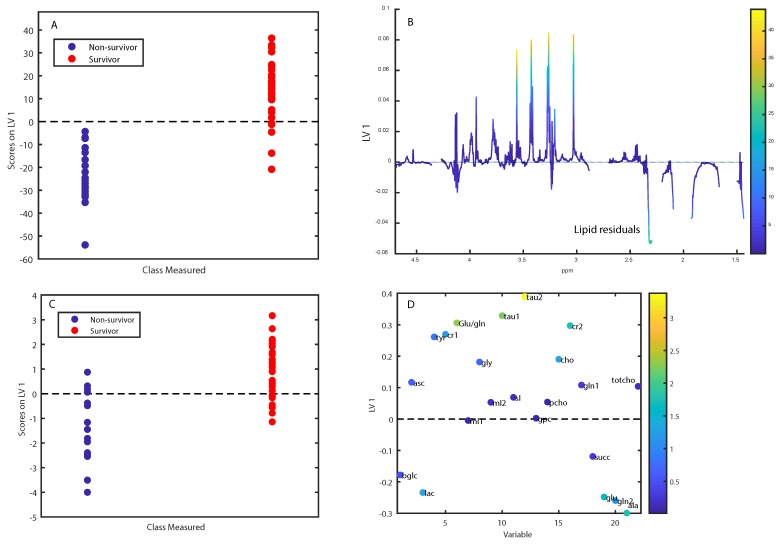
Score- and loading plots for separating 10 year survivors from non-survivors among patients who did not receive systemic adjuvant treatment. (**A**,**B**) show plots for prediction from spectral data, while (**C**,**D**) show plots for prediction from integrals. Labeled metabolites: beta-glucose (bglc), ascorbate (asc), lactate (lac), tyrosine (tyr), creatine (cr1), glutamate/glutamine (glu/gln), myo-inositol (mI1), glycine (gly), myo-inositol (mI2), taurine (tau), glycerophosphocholine (gpc), phosphocholine (pcho), choline (cho), creatine (cr2), glutamine (gln1), succinate (succ), glutamate (glu), glutamine (gln2), alanine (ala), total choline (totcho). The loadings are colored according to VIP-scores.

Five-year survival could not be predicted from spectral data, giving a classification accuracy of 57% (*p* = 0.18). Excluding samples with a high lipid content did not improve classification (accuracy = 56.0%, *p* = 0.25), neither did exclusion of the 24 patients who received adjuvant therapy (accuracy = 52.4%). Ten-year survival could be predicted from spectral data, however, only with a low accuracy (58.9%, *p* = 0.03), and this was reduced to 54.9% after removal of high-lipid samples. In patients who did not receive adjuvant therapy, spectral data could predict ten-year survival with an accuracy of 69.7% (Figure 5, *p* = 0.013). The score and loading plots show that lipid residuals comprised the most important discriminating metabolites, with non-survivors having more lipid signals and less metabolites. Using normalized integrals as prediction data to minimize the effect of lipid residuals gave a similar accuracy of 67.3%, with signals for lactate, glutamate, glutamine and alanine being higher in non-survivors (Figure 5C,D).

## 3. Discussion

In this study, we evaluated the feasibility of using a historical biobank for NMR metabolomics analyses. While historical biobanks provide unique opportunities to analyse samples with a long follow-up time and to assess prognosis in patients who did not receive adjuvant treatment, working with older biobanks is associated with possible pitfalls. We performed metabolomic and histologic analyses of tissue samples from a historical biobank, OsloVal, in order to investigate sample suitability for metabolomic analysis compared to an ongoing biobank.

Since metabolites are sensitive to degradation [9], variation in sample handling and storage could lead to loss of metabolic information. However, there are currently no reliable methods to determine whether this has occurred. RNases responsible for degrading RNA have temperature-dependent activity and low RIN values are therefore often the result of improper sample handling and storage [10]. Therefore, we hypothesized that the RIN could be used as an indicator of metabolic tissue degradation. The samples in the OsloVal cohort from before 1994 had a significantly lower RIN compared with Oslo2. The difference in RIN is expected to have been even more pronounced if RNA from Oslo2 had been isolated with the same method as used for OsloVal, as isolation by TRIzol generally yields lower RIN-values [11]. Interestingly, no difference in RIN could be found when comparing the samples in the most recent part of the OsloVal biobank with the Oslo2-cohort (RIN 6.45 and 6.30, respectively), supporting that the most recent samples were obtained and stored in accordance with modern standards. A low RIN was associated with higher levels of choline and GPC, and lower levels of creatine and Pcho. The metabolic profile of OsloVal was significantly different from Oslo2, with higher levels of choline, glycine, tyrosine, glutamate and glutamine, and lower levels of lactate and myo-inositol. Torrell et al. investigated the effect of extended thawing on tissue metabolite levels, and showed that for glycine, tyrosine, glutamate and glutamine, levels increased after thawing, while levels of myo-inositol and lactate decreased [12,13]. Choline was not measured in this referred study. In a study performed at our group by Haukaas et al., choline was found to increase with delayed freezing time of tissue samples, while ascorbate and creatine were found to decrease [9]. Furthermore, levels of glycine, choline, and tyrosine increased after prolonged experiment time, which we suggested could result from physical release of metabolites due to structural degradation. As the metabolite differences between OsloVal and Oslo2 are consistent with previous reports on the effect of thawing and delayed freezing, it is likely that a combination of chemical and structural degradation have occurred in OsloVal. Although variation in RIN is not exclusively caused by delayed freezing time, we suggest that RIN can serve as an indicator of metabolic degradation when using older biobank material for metabolomics studies. 

It has been demonstrated in multiple independent cohorts that metabolite data can predict presence of cancer tissue and ER-status in breast tumour samples with high sensitivity and specificity, and similar metabolomic differences have been consistently seen [5,14,15]. Whether this could be reproduced could therefore give an additional indication of the quality of the data. For prediction of cancer tissue versus non-cancer tissue, we achieved a classification accuracy of 74.6%, with cancer samples having less lipid residuals compared with non-cancer. While this is an expected result considering that normal breast tissue contains significant amounts of fatty tissue, previous studies have highlighted choline-compounds as discriminating metabolites, although different spectral regions were used [15]. For prediction of ER-status, a classification error of 63.1% was achieved. This is lower than what has been found in other cohorts, which generally have yielded classification accuracies approaching or exceeding 80% [5]. We found that ER+ samples had higher levels of creatine, phosphocholine and taurine, while ER- samples had higher levels of glycine and choline, which is in accordance to previous studies [5]. Additionally, we have previously observed higher levels of alanine and GPC in ER- samples, but these metabolites were not readily visible in our current loading plots. When classifying samples based on progesterone receptor status, we found that PR- samples had higher levels of lactate, glycine, GPC and choline, while having lower levels of taurinePcho and creatine. This is different from our previous study in which PR- samples had higher Pcho and creatine [5]. There may be several reasons for this discrepancy. Classification accuracies for determining PR status were lower than those of ER determination, both in this cohort and in the previous [16], which could result in less reliable loadings. Additionally, PR-status in the older part of OsloVal was determined by dextran-coated charcoal assay, which is a method no longer in use for this purpose. Considering that several samples changed ER-status after histological re-examination, this may also be the case for PR determination.

We have previously shown that tissue metabolomic profiles are associated with prognosis, particularly in ER+ breast cancer [8,14]. In this study, neither five- nor ten-year survival from the whole group or the ER+ samples as a subset could be predicted from metabolomic profiles. Because all patients who received chemotherapy also received hormonal therapy, we investigated possible associations between metabolomic profile and survival in the 50 patients who received surgery only. This may allow us to assess if metabolism reflects the natural aggressiveness of breast cancer in patients who do not receive adjuvant systemic treatment. While no relationship was found for five-year survival, ten-year survival could be predicted from spectral data with an accuracy of 69.7%. However, the score and loading plots showed that the main discriminating metabolites were lipid residuals, with non-survivors having more lipids. This may not reflect true metabolomic markers of aggressiveness but rather the lipid content of the biopsy, which may vary according to surgical technique and which area of the tumour is biopsied. To correct for this, we repeated the analysis using metabolite integrals. The classification of 10-year survivors and non-survivors remained significant, with an accuracy of 67.3% (*p* = 0.027). However, the metabolites which had higher levels in non-survivors, lactate, alanine, glutamate and glutamine, are significantly affected by adjacent lipid peaks. Therefore, the validity of the model remains uncertain.

The fact that the older portion of the OsloVal cohort was shown to have significantly lower RIN-values when compared to the Oslo2-cohort and that the cohorts show metabolic differences previously associated with tissue degradation suggests that loss of metabolic information occurred. Based on this work, some issues with using historical biobanks for metabolomic analyses of cancer tissue can be highlighted. The importance of standardized biobanking protocols should be emphasized. Before starting data analyses, it is important to obtain detailed descriptions of how the patients were selected for sampling, in order to assess the possibility of selection bias. Detailed documentation on how samples were handled immediately after resection and how they were stored in the years leading up to analysis is of crucial importance. If this information is lacking, measures of RNA integrity, such as RIN, could possibly be used as a surrogate marker for tissue integrity. It should be evaluated whether the clinical information obtained from the patients’ journals was determined using methods that allow comparison to more modern cohorts, exemplified here by determination of hormone receptor status. The need for histopathology to confirm the presence of tumour tissue should be considered, as exemplified by the fact that almost all the samples that were excluded because of low tumour content were from the older cohort. In conclusion, while older biobanks can provide valuable tissue samples as well as opportunities to study long-term prognosis, the use of such samples requires thorough quality assurance to ensure that the samples are suitable for metabolomics studies.

## 4. Materials and Methods 

### 4.1. Sample Materials

A total of 100 primary tumour samples from patients operated between 1983 and 1997 at Oslo University Hospital who had not received neoadjuvant chemotherapy were included in this study (The OsloVal cohort). The study was approved by the regional ethical committee (approval number 2010/498). The samples were gathered in two different time periods, 1983–1989 and 1994–1997, during which different sampling protocols were used. Samples gathered from 1994 to 1997 (*n* = 17) were taken from the tumour centre, frozen in liquid nitrogen quickly after surgery, then stored in −80 °C until the time of analysis. The remaining tumour samples from 1983–1989 (*n* = 83) were sampled using a less stringent sampling protocol, in which the samples may not have been immediately frozen and were sampled from an unknown location within the tumour. These samples were stored in −30 °C for several years before being moved to storage in −80 °C until the time of analysis. The frozen samples from both cohorts were cut into three pieces and the middle piece was sent for metabolomic analysis and the remaining tissue was used for gene expression analyses. 

Information about clinical stage and (ER- and PR)-status was collected from the patient journals. ER- and PR-status in samples collected before 1994 was determined by dextran-coated charcoal assay, while immunohistochemistry was used after 1994. We reassessed ER status for all the samples using immunohistochemistry, as described in the next sections.

For quality control, we compared the samples with samples from the ongoing Oslo2-biobank [3]. Clinical information relating to the samples used from this cohort is available from the cited reference. This cohort consists of primary breast carcinoma tissue samples from patients operated at Oslo University Hospital (Radium Hospital and Ullevål Hospital) in the time period 2006–2009. Patients gave written consent for participation in the research, and the study was approved by the regional ethical committee (approval number 2016/433). The samples were fresh frozen after surgery and stored in −80 °C. A piece from the tumour was cut into three parts. The middle section was used for metabolomic analyses (*n* = 228) and the remaining tissue was used for gene expression analyses. 

### 4.2. HR MAS MRS-Experiments

Metabolomic analyses were performed by high-resolution magic angle spinning magnetic resonance spectroscopy (HR MAS MRS) using a Bruker Avance DRX600 spectrometer (Bruker Biospin GmbH, Germany), equipped with a 1H/13C MAS probe. The tissue samples were analysed in accordance with the HR MAS MRS protocol described by Giskeødegård et al. [17]. Tissue samples (mean sample weight = 6.64 mg, SD = 2.30 mg) were cut to fit a disposable 30 µL insert, with an added 3 µL of D_2_O (99.8%) with sodium formate (HCOONa, 25mM). The samples were cut by scalpel to fit into a MAS insert quickly (<5 min) on a specially designed workstation cooled down with liquid nitrogen to keep the samples frozen during preparation. The samples were spun at 5 kHz at the magic angle. The samples were kept at 5 °C throughout data acquisition to minimize tissue degradation. Data acquisition time averaged at 89 minutes for all the experiments, with a standard deviation of 14 minutes, due to differences in time spent to achieve an acceptable shim. Spectral resolution was assessed during shimming by assessing the half-width of formate. A Carr-Purcell-Meiboom-Gill (CPMG) sequence was used, with an effective echo time of 77 ms, a spectral width of 20 ppm (−5 to 15 ppm), and 256 scans. 

### 4.3. Spectral Preprocessing

**Figure 6:**
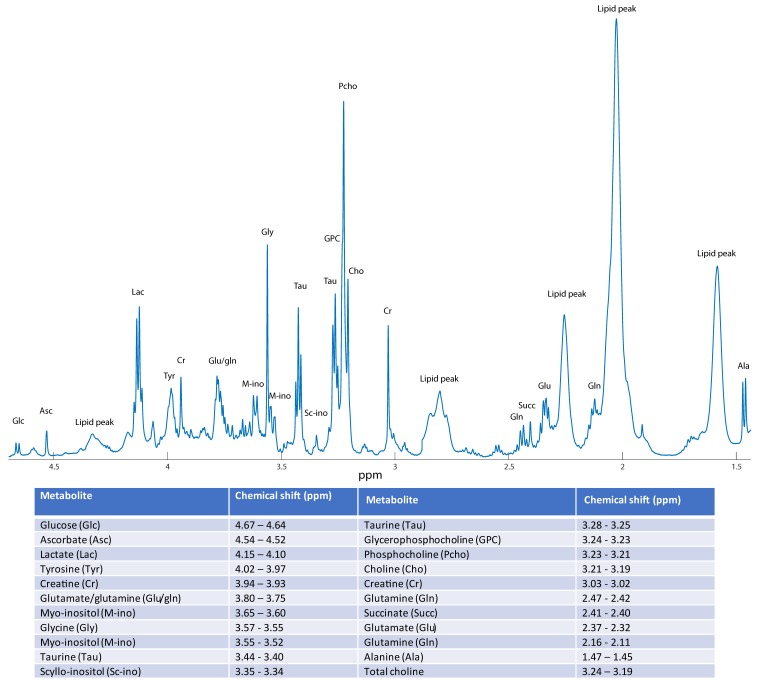
The mean OsloVal-spectrum after preprocessing, with metabolite annotations. The annotated lipid peaks were removed for the data analysis. Quantified metabolites with corresponding chemical shifts are shown in the table.

The free induction decays were Fourier-transformed into spectra consisting of 64,000 points, following 0.30 Hz line broadening. Phase correction was performed automatically in TopSpin 3.1. The spectra were then imported into MATLAB 2014a (The Mathworks, Inc., USA). The spectral region from 4.70 ppm to 1.40 ppm was selected for analysis. The spectra were shift-referenced to creatine at 3.03 ppm. The baseline was corrected by subtracting the minimum value in the spectrum from each point, making the lowest point in every spectrum equal to zero. Peaks were aligned using Icoshift [18]. Lipid peaks at 4.36–4.27, 2.88–2.70, 2.30–2.20, 2.09–1.93, and 1.67–1.50 ppm were excluded from the spectra and the spectra were mean-normalized by dividing each spectral variable with the average spectral intensity. Metabolite integrals were obtained by integrating the area under the peaks for all visible and identifiable metabolites in the pre-processed spectra, and re-normalizing them. The chemical shifts of the quantified metabolites and the mean spectrum after pre-processing are shown in Figure 6.

### 4.4. Histologic Examination

After analysis by HR MAS MRS, the samples were fixed in 10% formalin and embedded in paraffin blocks. A section of 5 µm was taken from the middle and stained with haematoxylin, eosin and saffron. The sections were examined for the presence of tumour tissue and samples without clear presence of tumour tissue were excluded from further analysis. ER-status was redetermined from adjacent tissue sections using immunohistochemistry with a cut-off of more than 1% ER-positive (ER+) tumour cells being considered ER+. A histologic evaluation was performed with guidance from an experienced breast cancer pathologist. For the metabolomic analyses, the redetermined ER-status from the analysed samples was used.

### 4.5. Molecular Genetic Analyses

Tissue adjacent to the sample used for metabolomic analysis was used for RNA microarray-analyses. For the OsloVal-samples, mRNA extractions were performed on the QIA symphony SP robot from Qiagen. An amount of 400 µL RLT buffer was added to the samples while on dry ice, followed by homogenization (Tissuelyser, Hilden, Germany). The QIA symphony RNA Kit cat# 931636 from Qiagen was used. For the Oslo2-samples, RNA was isolated with TRIzol (Invitrogen, Carlsbad, CA, USA). Extracted RNA was quantified using Nanodrop 1000 and the RNA integrity number (RIN) determined using the Agilent Bioanalyzer. The RIN was compared between samples from the two different time periods in the OsloVal-cohort, as well as with the Oslo2-cohort, in order to assess tissue integrity. 

### 4.6. Statistical Analysis

All spectral variables were mean centred before multivariate analysis. The data were examined for natural clusters and outliers by principal component analysis (PCA). Orthogonal partial least squares discriminant analysis (OPLS-DA) was used to classify samples according to clinical and prognostic factors. OPLS-DA is a variation of PLS-DA which simplifies model interpretation by capturing the predictive information in the first component and the remaining components describe variation orthogonal to the first component. Validation was done by 10-fold cross-validation over 20 iterations. The average classification error was plotted against an increasing number of components and the first local minimum in the cross-validation plot was chosen as the optimal number of components. Model significance was assessed by permutation testing in which the model data were shuffled to give random classification. This was repeated 1000 times and the *p*-value is equal to the percentage of permutated models producing a classification error better than or equal to the model being tested. RIN and FWHM were compared using Wilcoxon rank sum-tests. When comparing RIN-values, all 100 samples from the OsloVal-cohort were used, along with all 395 samples from the Oslo2-cohort.

## Figures and Tables

**Table 1:** Clinical cohort description for OsloVal.

OsloVal-Cohort Description, *n* = 74	
Age	57.7 (33–85)
ER-status	49 ER+, 25 ER-
PR-status	40 PR+, 30 PR-, 4 unknown
Grade (1-3)	8 (Grade 1), 28 (Grade 2), 24 (Grade 3), 14 (Unknown)
T-stage	30 (T1), 29 (T2), 5 (T3), 6 (T4), 4 (Unknown)
N-stage	40 (N0), 21 (N1), 5 (N2), 6 (N3), 2 (Unknown)
Adjuvant chemotherapy *	24 (Yes), 50 (No)
Endocrine therapy *	24 (Yes), 50 (No)
Five year survival	68%
Ten year survival	54%

Abbreviations: ER = Oestrogen receptor, PR = Progesterone receptor. * All patients who received chemotherapy also received endocrine therapy, and vice versa.

## Data Availability

The data from OsloVal are publicly available on MetaboLights with the identifier MTBLS1355.
